# A Common Drug With a Dangerous Side Effect: Acute Rhabdomyolysis Caused by the Synergistic Effect of Isotretinoin and Exercise in an Adolescent

**DOI:** 10.7759/cureus.10929

**Published:** 2020-10-13

**Authors:** Kingshuk Dasgupta, Peter Lim, Hannah Reedstorm

**Affiliations:** 1 Department of Pediatrics/Pediatric Critical Care, Sanford School of Medicine of the University of South Dakota/Avera McKennan Children’s Hospital & University Health Center, Sioux Falls, USA; 2 Department of Pediatric Infectious Diseases, University Hospitals (UH) Cleveland Medical Center - Rainbow Babies and Children’s Hospital, Cleveland, USA; 3 Pharmacy, Avera McKennan Children’s Hospital & University Health Center, Sioux Falls, USA

**Keywords:** rhabdomyolysis, isotretinoin adverse effects, accutane, creatine kinase, acne, exercise, musculoskeletal system, isotretinoin, myoglobin, adolescent

## Abstract

Isotretinoin or 13-cis-retinoic acid, a derivative of vitamin A, is a highly effective therapy for severe and resistant acne. Its usage is restricted worldwide because of its teratogenic potential. The most common side effects are dermatologic, cheilitis, increased skin fragility, and susceptibility to sunburns. Rare side effects include myalgias and arthralgias. It also causes transient laboratory abnormalities such as elevated creatine kinase (CK). Very few cases of isotretinoin-induced severe muscle injury leading to rhabdomyolysis have been reported in the literature. We report a 15-year-old adolescent who developed rhabdomyolysis after a bout of vigorous exercising while on long-term isotretinoin therapy.

## Introduction

What we know

Isotretinoin (Accutane) is a safe, proven, and highly efficacious therapy for resistant acne [1-5]. Isotretinoin has several side effects that are relatively minor and usually resolve quickly following cessation of therapy. There have been several reports of isotretinoin-associated muscle injury resulting in elevated creatinine kinase (CK) levels [6-11]. Surprisingly, only a few cases of isotretinoin-associated rhabdomyolysis have been reported in relevant literature to date [10-12]. Rhabdomyolysis is a medical emergency and the leading cause of acute kidney injury, which can be fatal [13-14]. We present the case of a 16-year-old female on long-term isotretinoin therapy for recalcitrant acne who developed rhabdomyolysis after an episode of vigorous exercise.

## Case presentation

A 16-year-old Caucasian female with recalcitrant acne was brought to the emergency room for significant bilateral hip pain after a strenuous stationary bike workout one day prior to presentation. She had done biking workouts before but never as vigorously. She recalled taking three ibuprofen tablets (200 mg) and two hydrocodone tablets (5 mg) two times each prior to presenting herself to the emergency room (ER). On the day of admission, she started experiencing thigh soreness out of proportion to her usual post-exercise soreness along with lower extremity weakness. She also noticed a darkening of her urine. She was started on isotretinoin 1 mg/kg/day approximately six months prior to the onset of her symptoms by her dermatologist.

On physical examination, she had severe, bilateral thigh tenderness, which worsened with passive movement. She rated her leg pain a 6 out of 10. She was unable to walk without support. She denied any chest pain, shortness of breath, nausea, emesis, diarrhea, or pain elsewhere. She denied any history of intramuscular injections. Laboratory workup revealed a significantly elevated creatinine kinase (CK) of 32,406 U/L (normal 20-180 U/L). She was being followed closely by her dermatologist as an outpatient and her CK was 59 U/L one month prior to this event.

Further laboratory workup was notable for myoglobinuria and hemoglobinuria (3+ blood in the absence of red blood cells (RBCs)). Urine myoglobin was 14 mg/L (normal: 0-1 mg/dL) and urine pH was 6. Her liver enzymes were elevated with alanine transaminase (ALT) of 120 U/L (N 9-23U/L) and aspartate aminotransferase (AST) of U/L (Normal: 17-33U/L). Her kidney function was normal with a blood urea nitrogen (BUN) of 15 mg/dL (9-22 mg/dL) and creatinine 0.8 mg/dL (0.3-0.6 mg/dL). Based on her laboratory results, she was diagnosed with rhabdomyolysis and was immediately admitted. Isotretinoin was discontinued and she was treated aggressively with intravenous fluids with added sodium bicarbonate (150 mEq/L) to alkalinize her urine. Serial monitoring of CK levels and renal function were performed. Her CK decreased daily and was 2290 U/L on Day 5 of admission (Table 1). Her serum creatinine remained within normal limits throughout her hospital stay. She regained her motor strength after working with physiotherapy and was walking normally with no pain at the time of discharge (hospital day five). AST was 364 U/L and ALT was 144 U/L at the time of discharge. The liver ultrasound and coagulation profile were normal. Her CK after one month of discharge was 239 U/L, and she was asymptomatic.

**Table 1:** Laboratory values HCT: hematocrit; WBC: white blood cell; PT: prothrombin time; PTT: partial thromboplastin time; INR: international normalized ratio

Hb	11.5-15.5 g/dL	13.1				
Hct	35-45	39.6				
WBC	4.5-13.5 K/uL	11				
Platelet	140-440 K/uL	277				
PT	11.7-15.1s	12.7				
PTT	32-44 s	30				
INR	1.2	0.9				
Sodium	138-146 mmol/L	134				141
Potassium	3.4-4.7 mmol/L	3.5				4.2
Chloride	97-107 mmol/L	103				108
Blood urea nitrogen	9-22 mg/dL	15				9
Creatinine	0.3-0.6 mg/dL	0.8				0.6
Calcium	9.2-10.5 mg/dL	9.1				9.5
Glucose	70-99 mg/dL	94				
Creatinine kinase		32406	27903	15324	5914	2290
Aspartate aminotransferase	17-33 U/L	330	405	364	315	304
Alanine aminotransferase	9-23 U/L	104	119	164	150	144
Laboratory values	Reference values	Day 1	Day 2	Day 3	Day 4	Day 5

## Discussion

Rhabdomyolysis is characterized by generalized muscle pain, fatigue, weakness, and a >5-fold increase in serum CK levels and myoglobinuria [14]. If untreated, rhabdomyolysis can lead to acute renal failure due to the obstruction of renal tubules by the precipitated myoglobin [15]. Rhabdomyolysis is a medical emergency and can be fatal [15]. Acute rhabdomyolysis can result from traumatic or non-traumatic causes. Overall, the most common cause of rhabdomyolysis is muscular trauma; other non-traumatic causes include vigorous exercise, infections, metabolic myopathies, hypothermia, toxins, and drugs [14]. Non-traumatic rhabdomyolysis is more difficult to diagnose. The management of rhabdomyolysis is supportive and involves the prevention of all the factors that can precipitate acute renal failure such as volume depletion.

Oral isotretinoin, a retinoid and synthetic analog of vitamin A, is thought to work by inhibiting sebaceous gland function and keratinization. It is a highly effective therapy for resistant acne and has been used since 1976 for the treatment of severe nodulocystic acne and acne unresponsive to conventional therapy [1-4]. The approved dose is 0.5 to 1 mg/kg/day given with food in two divided doses for adolescents >=12 years of age. A normal treatment course is usually 15 to 20 weeks.

Isotretinoin has several well-documented side effects. In fact, one or more side effects can be routinely expected to occur in most patients. Most of the time, these side effects are predictable, resembling those from the parent compound’s syndrome of chronic hypervitaminosis A. They are relatively minor, comprising of mucocutaneous effects (90%), such as cheilitis, xerosis, hair loss, and sun hypersensitivity reactions. Fortunately, these are rapidly reversible after the discontinuation of the drug [1,7].

The distribution of isotretinoin is restricted because of teratogenic effects and a centralized risk management program called iPLEDGE [16] is used to minimize pregnancy exposures. Additionally, musculoskeletal side effects, such as pain, tenderness, or stiffness of muscles, have also been reported commonly (16-51%). However, true myopathy is rarely observed [17]. Patients with musculoskeletal symptoms may or may not have concomitant elevation in CK levels.

Moreover, a mild increase in CK (with or without muscle-related symptoms) has been frequently noted in patients on isotretinoin therapy [10-11]. For instance, elevated CK <3 times the normal was recorded at least once during isotretinoin therapy in 165/442 (37.3%) patients in one study [11]. Minor elevations in CK during isotretinoin therapy is considered a benign phenomenon [11].

HyperCKemia >5,000 IU/L occurs much more rarely (only 6/442 patients in the same study) with isotretinoin treatment [6,8,11,18]. Some cases have been reported in the context of concomitant vigorous physical exercise [11,18-19]. HyperCKemia seems to occur within the first weeks of maximal isotretinoin dosage. The intensity of exercise that leads to HyperCKemia is unknown. High CK may be indicative of muscle membrane damage, which may also cause the release of other muscle proteins, such as myoglobin, into the bloodstream. In this setting, it is reasonable to assume that if muscle damage is extensive, large amounts of myoglobin released from the damaged muscles could lead to kidney tubular damage, precipitating acute kidney injury. The damaged muscle itself is capable of completely regenerating.

To date, only two cases of isotretinoin-associated rhabdomyolysis have been reported in the English literature. Both patients recovered quickly and completely following the cessation of the drug [10,12]. Additionally, one fatality from isotretinoin-associated rhabdomyolysis and multiorgan failure has also been reported postmortem [13]. Like the previously reported cases, our patient had performed vigorous exercise while on isotretinoin. Her rhabdomyolysis started approximately six months after being on isotretinoin, which is much later than the other two reported patients (five weeks in both). Interestingly, our patient is only the second patient who had demonstrable myoglobinuria and the first patient who had urine myoglobin quantified in addition to HyperCKemia in this clinical setting [12]. She had a normal CK one month prior and her symptoms and elevation in CK levels started after vigorous exercise, demonstrating that exercise is an independent risk factor for HyperCKemia for patients on isotretinoin. Our patient did not progress to acute kidney injury and recovered with supportive treatment with no sequelae. Her CK was still elevated one month later but was markedly lower.

## Conclusions

Isotretinoin is a widely prescribed drug for acne. This paper adds to the relatively sparse body of evidence regarding the synergistic effect of exercise and isotretinoin usage in causing HyperCKemia and, very rarely, rhabdomyolysis. The case also demonstrates the presence of myoglobinuria as a marker of muscle injury and rhabdomyolysis in this setting and that synergism between exercise and Isotretinoin usage can lead to both early and late-onset HyperCKemia and rhabdomyolysis. We suggest that routine measurements of serum CK levels and any muscle complaints should be closely followed in exercising patients on isotretinoin throughout the duration of therapy. Mild to moderate exercise seems to be well-tolerated; however, patients should be counseled to abstain from vigorous exercise/activity for the duration of isotretinoin treatment. Patients on isotretinoin therapy should promptly seek medical evaluation for any new musculoskeletal complaints while on isotretinoin therapy.
